# A multicenter mixed-methods study on the effects of intermittent fasting in patients with immune thrombocytopenia receiving thrombopoietin receptor agonists

**DOI:** 10.3389/fnut.2025.1434484

**Published:** 2025-02-26

**Authors:** Mohamed A. Yassin, Muna AlRasheed, Taghreed Al-Eisa, Noura Alhashim, Fiasal Alsayegh, Tarek E. Abouzeid, Mohamed Abd El Fattah, Maryam Alfili, Neveen Shalaby, Abdullah Alotaibi, Nourah Aljuwaisri, Anwar Almasbahi, Rii Saleeb, Hend Abdelaziz, Awni Alshurafa, Omar Ismail, Rola Ghasoub

**Affiliations:** ^1^Department of Hematology and Bone Marrow Transplant, National Center for Cancer Care and Research, Hamad Medical Corporation, Doha, Qatar; ^2^College of Medicine, Qatar University, Doha, Qatar; ^3^Department of Hematology, Adan Hospital, Ministry of Health, Kuwait, Kuwait; ^4^Al-Jahra Hospital, Ministry of Health, Kuwait, Kuwait; ^5^Department of Hematology, Mubarak Alkabeer Hospital, Ministry of Health, Kuwait, Kuwait; ^6^College of Medicine, Kuwait University, Kuwait, Kuwait; ^7^Internal Medicine Department, Almouwasat Hospital, Dammam, Saudi Arabia; ^8^Accsight, Jeddah, Saudi Arabia; ^9^Kuwait Oil Company, Ahmadi Hospital, Kuwait, Kuwait; ^10^Department of Internal Medicine, Hematology Unit, Adan Hospital, Kuwait, Kuwait; ^11^Department of Internal Medicine, Hematology Unit, Faculty of Medicine, Tanta University, Tanta, Egypt; ^12^Department of Hematology, Sabah Hospital, Kuwait, Kuwait; ^13^Department of Hematology Unit, Al Amiri Hospital, Kuwait, Kuwait; ^14^Department of Hematology Unit, Jaber AlAhmad Hospital, Kuwait, Kuwait; ^15^Department of Hematology, Amiri Hospital, Kuwait, Kuwait; ^16^Clinical Hematology Department, Al Jahra Hospital, Ministry of Health, Kuwait, Kuwait; ^17^Department of Pharmacy, National Center for Cancer Care and Research, Hamad Medical Corporation, Doha, Qatar

**Keywords:** Immune thrombocytopenia, thrombopoietin receptor agonist, avatrombopag, eltrombopag, romiplostim, fasting, intermittent fasting

## Abstract

**Introduction:**

In recent years, significant advances have been made in the treatment of immune thrombocytopenia (ITP) with the development of thrombopoietin receptor agonists (TPO-RAs). TPO-RAs are often used following the failure of prior therapies or when bleeding episodes persist despite glucocorticoid use. In Muslim countries, where religious observance includes 16/8 intermittent fasting, the timing of medication administration may be affected. This study is the first to evaluate the impact of Ramadan fasting on patients receiving different TPO-RAs.

**Methods:**

A multicenter mixed-design study was performed in which Muslim patients who fasted during Ramadan while receiving TPO-RAs were interviewed between 2015 and 2023. Patient responses before, during, and after Ramadan were evaluated retrospectively. The bleeding tendency was assessed as (1) no bleeding, (2) minor cutaneous/mucosal bleeding, or (3) severe bleeding that involves major organs.

**Results:**

The present study included 100 patients from three Muslim countries, including Qatar, Kuwait, and Saudi Arabia, across four tertiary centers. A complete response was observed in 63% of patients on ROM, 46% on ELT and 37% on AVA. For AVA, the mean platelet (PLT) count before Ramadan was estimated at [146.11 ± 111.76], while during Ramadan, it dropped to [131.7 ± 107.6]. For patients on ELT, the mean PLT count before Ramadan was estimated at [120.02 ± 59.7], while during Ramadan, it dropped to [100.8 ± 68.16] (*p* = 0.016). For patients on ROM, the mean platelet count before Ramadan was estimated at [122.68 ± 80.57], while during Ramadan, it was [130.94 ± 84.96]. Only 3% (3 patients on ELT) experienced bleeding episodes.

**Conclusion:**

This study supports the feasibility of Ramadan fasting for ITP patients receiving TPO-RAs. Further studies with a larger sample size are recommended to investigate the impact of other types of fasting on the efficacy and safety of TPO-RAs.

## Introduction

In recent years, the diagnosis and management of ITP have become more explicit due to the standardization of definitions and terminology according to the consensus reached by the International Working Group (IWG) (1). Moreover, there have been significant advances in treating ITP, including developing novel therapies and refining clinical practice guidelines (2, 3). The American Society of Hematology and International Consensus guidelines recommend using thrombopoietin receptor agonists (TPO-RAs) as the preferred second-line therapy for ITP patients after first -line treatment failure. (2, 3). Currently, three TPO-RAs are approved by the Food and Drug Administration (FDA) for use in adult patients with primary ITP who are refractory to first-line therapies: romiplostim (ROM), approved as a once-weekly subcutaneous injection; eltrombopag (ELT); and avatrombopag (AVA), approved as a once-daily oral treatment (4–6). When selecting TPO-RAs, different factors should be considered, such as route of administration, dietary restrictions, drug interactions, and cost (7). For optimum absorption, ELT should be taken at least 2 h before or 4 h after using antacids and dairy products owing to chelation (4). In contrast, ROM and AVA have no such restrictions. Moreover, ROM is the only parenteral TPO-RA that may be suitable for patients with gastrointestinal impairments (5, 6). Therefore, drug administration is crucial in patients with ITP.

Intermittent fasting (IF) is becoming popular as an effective method for caloric restriction. According to the International Food Information Council (IFIC), IF is cited as one of the most frequent dietary patterns in 2022 in the United States of America (USA) (8). Different IF strategies exist, including alternate-day fasting, a fasting-mimicking diet (FMD), a 24-h fast, time-restricted feeding (TRF), and the 16/8 method (9). Several studies have reported valuable beneficial outcomes and the safety of IF, including hypertension, weight reduction, diabetes, cancer, and other diseases (10–14). In addition, several benefits were documented in patients with autoimmune diseases, including lowering serum leptin levels, which improves regulatory T cells and lowers inflammation, as well as lowering oxidative stress and inflammatory markers, which help manage symptoms of diseases like rheumatoid arthritis (15–17). Additionally, reducing inflammation and modifying immunological responses has demonstrated the ability to enhance clinical outcomes in conditions like systemic lupus erythematosus (18).

Islamic fasting, specifically Ramadan fasting, is an IF strategy practiced widely in Muslim countries during the holy month of Ramadan (19). The fasting period is inconsistent depending on the geographical location’s latitude and the timing of the month of Ramadan during the seasonal cycle. Furthermore, Islamic fasting does not allow water consumption. Therefore, the stress on the physiological system during Islamic fasting differs from that of contemporary non-Islamic fasting (20).

With the different changes in fluid balance and hydration status observed during Ramadan, there is a notable increase in plasma osmolality (21). This physiological shift can lead to further disturbances in electrolyte levels, which may impact renal function by reducing the estimated glomerular filtration rate (eGFR) and serum creatinine (22). However, these findings were inconsistent and variable according to different factors such as the hydration level during non-fasting hours, the fluid consumption habits before fasting, and the presence of comorbidities such as diabetes and chronic kidney disease (23, 24). Moreover, the metabolic rate and insulin responses could be affected by the rapid consumption of high-caloric meals during a short period (20, 21). In comparison, non-Islamic IF practices generally allow for more flexible eating periods and may not involve the same level of fluid restriction or daily eating patterns, potentially resulting in different metabolic effects.

The annual observance of Ramadan through fasting by Muslims worldwide has not been comprehensively studied in terms of its health consequences. Therefore, it is important to consider the potential impacts of IF and alternative eating patterns on medication absorption. The question of whether patients with ITP should adjust their treatment plans because of fasting is one of the most posed inquiries. Amending a drug regimen may be required in some patients. Furthermore, food–drug interactions can alter drug bioavailability. To date, no guidelines or standardized protocols can help physicians correctly answer ITP patients regarding the feasibility of fasting during Ramadan. As such, the present study was designed to fill in this knowledge gap and to comprehensively investigate the impact of an intermittent fasting regimen (Ramadan fasting) on the administration of TPO-RAs in patients with ITP.

## Methods

### Study design

A multicenter, non-interventional, mixed-design study was performed in which we recruited Muslim patients who fasted during Ramadan while receiving TPO-RAs in Qatar, Kuwait, and Saudi Arabia at four tertiary cancer centers between 2015 and 2023. The duration of fasting was approximately 12 h from sunrise to sunset (the time of abstinence from food) during a 30-day period. Investigators collected patients’ data retrospectively, including complete blood count (CBC), platelet (PLT) count, and bleeding episodes, at three separate assessment points (before, during, and after Ramadan) using a standardized patient form. Electronic patient records were the source data used to complete the data collection form. Fasting status was confirmed by telephone calls to each patient. Platelet counts were obtained before and after the holy month of Ramadan and at least once in the middle of the month for all patients. The blood samples for the CBC were collected during routine follow-ups, with samples taken in the morning according to the laboratory’s scheduled hours. Additionally, efforts were made to identify any missing variables that could be obtained from alternative sources, such as physician notes or by contacting the treating physicians.

### Sampling and study population

We included all individuals aged 18 years and older with ITP who fasted during Ramadan. Pregnant women and those who were unavailable for blood measurements after Ramadan were excluded. The patients were screened as Muslim or non-Muslim after obtaining institutional review board (IRB) approval. Patients were then retrospectively asked over the phone using a pre-prepared script and confirmed that they were fasting during the month of Ramadan, and that process was documented. Patients were contacted and assessed for compliance with medication and proper timing of administration. The sample size was based on feasibility and practical considerations rather than statistical considerations. We expected to recruit 105 patients, divided into 35 patients on AVA, 35 on ELT, and 35 on ROM. To minimize potential selection bias, the patient selection criteria were designed to allow the enrollment of most patients at the participating sites.

### Objectives and outcomes measurement

The primary objective of this study was to evaluate the effect of Ramadan fasting on ITP patients receiving different TPO-RAs. The primary endpoints included a significant decrease in PLT count, bleeding tendency, and weighted mean PLT count. We used the IWG response criteria to evaluate patient responses (1). A significant drop in platelet count was defined as a drop in platelet count that is more than or equal to half of the baseline. The bleeding tendency was evaluated as either no bleeding, cautious bleeding, mucocutaneous bleeding, major bleeding in the central nervous system (CNS), gastrointestinal (GI), genitourinary, or life-threatening bleeding involving major organs (1). The secondary endpoints included the mean change in platelet count during the fasting period and laboratory findings by evaluating PLT number and mean platelet volume (MPV). As ELT absorption is influenced by food intake, the patients receiving ELT were asked about their routine administration of the drug, specifically in relation to the timing and type of meals during Ramadan. A standardized telephone script was used for all telephone interviews, focusing on specific questions regarding Ramadan fasting to enhance consistency and reliability in data collection. The interviews were conducted within a short time frame after Ramadan to improve recall accuracy. The investigator was responsible for obtaining approval of the study protocol, possible amendments, written patient information, informed consent, and assent forms from the independent ethics committee (IEC). All participants contacted by telephone interviews provided verbally informed consent before participating in the study. This study was conducted in compliance with the study protocol and applicable regulatory requirements and in accordance with the ethical principles of its origin.

### Statistical analysis

All continuous variables were summarized using descriptive statistics, including the mean, standard deviation, and minimum and maximum values. Categorical variables were summarized in frequency tables, showing the percentages of observed responses in each category. An independent t-test and ANOVA were used to compare quantitative and qualitative variables. The bleeding tendency was also evaluated as no bleeding, minor cutaneous and mucosal bleeding, or life-threatening bleeding involving major organs (i.e., the central nervous system, gastrointestinal system, or genitourinary system). Formal statistical hypothesis testing was not conducted. All statistical analyses were performed using the Statistical Analysis Software (SAS) System (SAS Institute, Cary, NC), version 9.2.

## Results

The present study included 100 patients from three Muslim countries: Qatar, Kuwait, and Saudi Arabia, in four tertiary centers. Forty-three subjects (43%) were males, whereas fifty-seven (57%) were females. The mean age was 42 years (range: 18–84). The demographics of the participants are summarized in Table 1.

**Table 1:** Demographics and baseline characteristics.

Variable	Total (*n* = 100)
Age, median (range), years	38 (18–84)
Gender, (n)	
Female	57
Male	43
Ethnicity, (n)	
Arab	96
Non-Arab (Asians)	4
Comorbidities, (n)	
Yes (Hypertension)	11
No	89
Splenectomy, (n)	
Yes	–
No	100
Prior ITP medications, (n)	
Steroids	100
IVIG	100
Rituximab	19
Previous major bleeding episodes, (n)	
Yes	2
No	98
TPO-RAs, (n)	
AVA	20
ELT	52
ROM	19

ITP, Immune thrombocytopenia, IVIG, Intravenous immunoglobulin AVA, Eltrombopag, ELT, Eltrombopag, ROM, Romiplostim.

The present study observed significant changes among the three products in platelet responses due to disturbed meal schedules. The proportion of patients who achieved a complete (PLT > 100 × 10^9^/L) and a partial response (PLT 50–100 × 10^9^/L) among the three products was 47 and 17%, respectively. A complete response was observed in 63% of patients on ROM, 46% of patients on ELT and 37% of patients on AVA, respectively. For AVA, the mean platelet count before Ramadan [146.11 ± 111.76], which was decreased to [131.7 ± 107.6] during Ramadan. However, the difference before and after Ramadan was statistically insignificant [146.11 ± 111.76] vs. [134.17 ± 60.9]. For patients on ELT, the mean platelet count before Ramadan was [120.02 ± 59.7], which dropped to [100.8 ± 68.16] during Ramadan. Similarly, the difference before and after Ramadan was insignificant [120.02 ± 59.7] vs. [136.04 ± 74.86].

**Figure 1:**
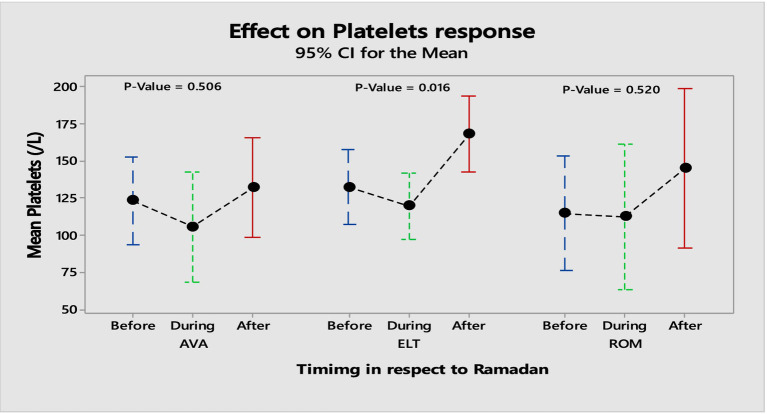
Collective significance of TPO-RAs platelet response with respect to timing.

**Figure 2:**
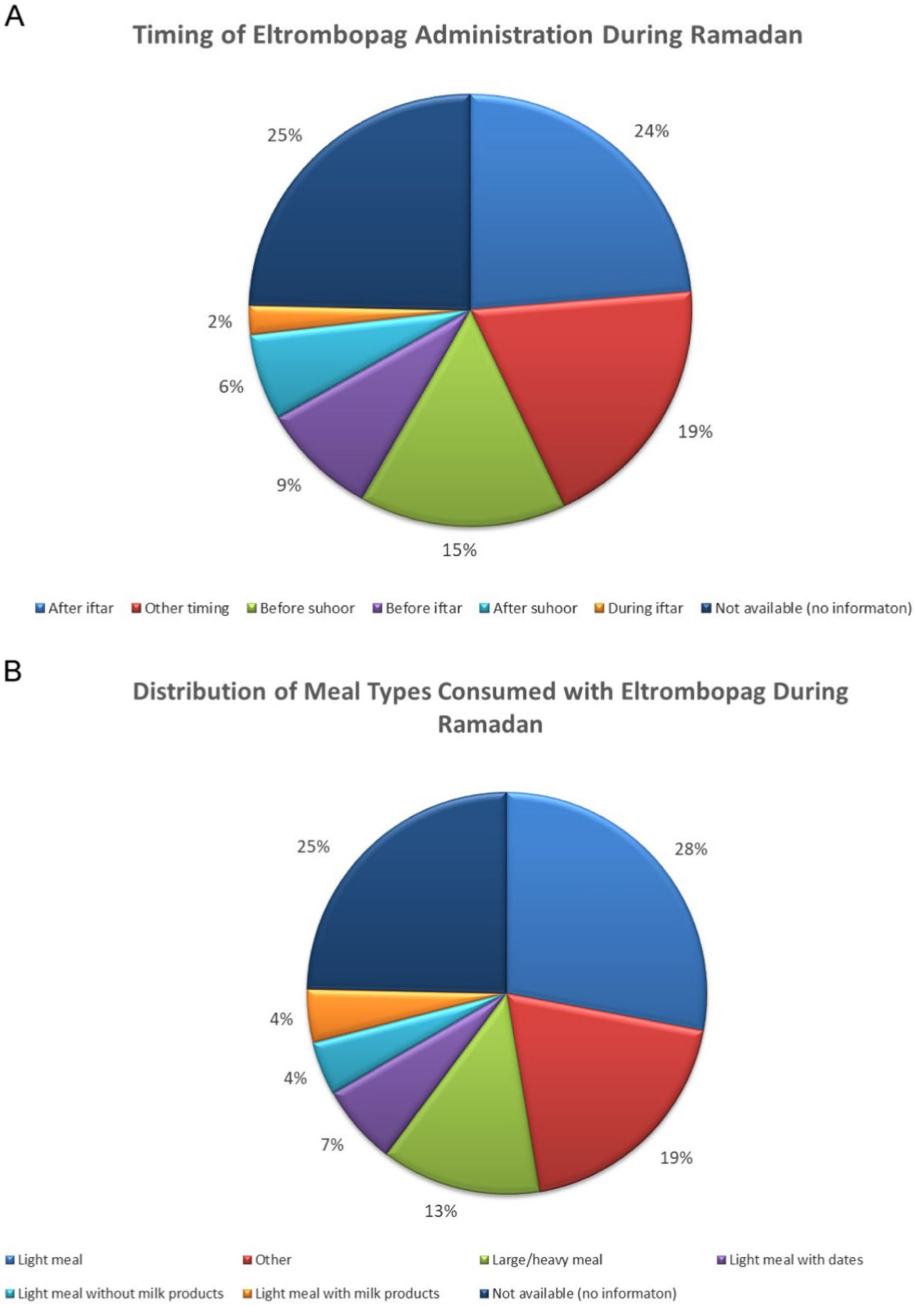
**(A,B)** Distribution of ELT administration times and the types of meals consumed during Ramadan.

For patients on ROM, the mean platelet count before Ramadan was [122.68 ± 80.57], which increased slightly to [130.94 ± 84.96] during Ramadan. While the difference in platelet count before and after Ramadan was statistically insignificant, there was a notable increase [122.68 ± 80.57] vs. [212.0 ± 113.72]. Table 2 summarizes the effects of the three TPO-RAs on PLT and MPV measured at three time points: before, during, and after Ramadan. Additionally, there was no significantcorrelation between sex, age, hypertension, MPV, and the baseline PLT counts with the response to the three TPO-RAs. Figure 1 illustrates the differences in the mean platelet counts in TPO-RAs concerning timing with respect to Ramada. Only 3% (3 patients on ELT) of those who fasted during Ramadan experienced bleeding episodes. Two patients experienced minor bleeding (ecchymosis and gingival bleeding), and one had a major bleeding episode (left hemorrhagic infarction). The platelet counts in these patients were 46 × 10^9^/L, 29 × 10^9^/L, and 4 × 10^9^/L, respectively. For patients on ELT, a distinct preference for administering the drug during the evening meal periods was observed, with the majority of doses taken after Iftar. Regarding meal types, light meals were the most commonly consumed with ELT. Figures 2 A,B show the distribution of ELT administration times and the types of meals consumed during Ramadan based on the available data from 35 patients.

**Table 2:** Comparison of platelet count and mean platelet volume across treatments.

Parameter	Mean	95% CI	
		Lower	Upper
Avatrombopag			
PLT before Ramadan	146.11	88.65	203.58
MPV before Ramadan	11.00	10.35	11.65
PLT during Ramadan	131.70	76.38	187.03
MPV during Ramadan	10.68	9.96	11.39
PLT after Ramadan	134.17	102.84	165.50
MPV after Ramadan	10.0143	8.67	11.35
Eltrombopag			
PLT before Ramadan	120.02	102.86	137.17
MPV before Ramadan	10.08	9.51	10.66
PLT during Ramadan	100.80	80.79	120.82
MPV during Ramadan	9.52	8.86	10.18
PLT after Ramadan	136.04	114.06	158.02
MPV after Ramadan	11.04	10.41	11.67
Romiplostim			
PLT before Ramadan	122.68	83.84	161.52
MPV before Ramadan	10.80	9.72	11.88
PLT during Ramadan	130.94	89.99	171.90
MPV during Ramadan	7.88	5.47	10.30
PLT after Ramadan	212.00	155.44	268.55
MPV after Ramadan	10.95	10.11	11.78

PLT, Platelets (×109/L); MPV, Mean platelets volume (fL); CI, Confidence interval.

## Discussion

This is the first study to evaluate the impact of Ramadan fasting in ITP patients receiving TPOR-RAs, which is of considerable relevance given the recent popularity of investigating IF in different settings. Despite the presence of evidence supporting the positive effects of IF on health and its potential application to various diseases (12–18), there are currently no established guidelines to help physicians respond to ITP patients who express a desire to fast while taking TPO-RAs. All Muslims practice fasting during the month of Ramadan. The month consists of 29–30 days yearly, in which Muslims fast for 12–16 h from dawn to sunset daily, depending on their geographical location (19). Islamic fasting shares similarities with time-restricted fasting; however, it differs in that there is no limitation on the consumption of energy or calories (19).

The findings from studies exploring the impact of Ramadan fasting on hematological indices have been contradictory. Al Hourani et al. reported a notable decrease in platelet count among 57 healthy female subjects in Jordan during Ramadan fasting, while no substantial variations were detected in hemoglobin, hematocrit, and red blood cell count values before and during the fasting period (25). A recent study investigated the impact of Ramadan fasting on patients with sickle cell disease showed a significant reduction in PLT and reticulocyte counts with Ramadan fasting (26). Nevertheless, no studies have examined the impact of fasting on ITP patients.

The exact mechanism by which IF fasting can affect platelet counts remains unclear, though multiple theories have been proposed in the literature. Ramadan fasting disrupts circadian rhythms, potentially altering the release of hormones like cortisol, insulin, and catecholamines, which impact platelet function, coagulation, and fibrinolysis (27). The observed reduction in platelet aggregation sensitivity to adenosine 5′-diphosphate (ADP), collagen, and adrenaline may directly result from these hormonal changes, modulated by their circadian fluctuations during fasting (28). Moreover, inflammatory mediators also play a role in platelet aggregation. With fasting, the lower levels of pro-inflammatory cytokines like TNF-*α* or interleukin-6 could explain the decreased platelet sensitivity to aggregation agents (29, 30). Additionally, the changes in nutrient intake during Ramadan, particularly in the absence of food for prolonged periods, may lead to changes in blood lipids, glucose, and other metabolic factors (31). While IF and prolonged fasting have beneficial impacts on metabolism, insulin sensitivity, and weight control, the effect on platelet counts appears to be complex and heterogeneous. In people with metabolic syndrome, which is associated with several conditions, including obesity, insulin resistance, and chronic inflammation, fasting has been shown to influence platelet function and production in different ways (28). High blood sugar levels can increase platelet activity through non-enzymatic glycation of platelet surface proteins, which occurs in hyperglycemic conditions. This glycation process diminishes the fluidity of the platelet membrane and heightens the likelihood of platelet activation (32). Additionally, hyperglycemia can intensify platelet reactivity through the osmotic effects of glucose, serving as a secondary mechanism (28, 32).

TPO-RAs have demonstrated comparable efficacies and satisfactory safety profiles in clinical trials. However, they differ in pharmacodynamic and pharmacokinetic properties. For example, ROM is administered subcutaneously; however, AVA and ELT are administered orally, rendering them vulnerable to food-drug interactions (4–6). This presents a key challenge in oral administration, as food can influence drug release, absorption, distribution, and metabolism (33). Additionally, fasting has been identified as a factor that impacts drug metabolism and response (34). In our study, we observed a significant difference in platelet count during Ramadan compared to that after fasting, with the platelet count being lower during the fasting period. However, the mean platelet level did not fall within the range of severe thrombocytopenia. Only one patient developed severe bleeding and required hospital admission (left hemorrhagic infarction).

Considerations for drug administration and drug interactions are essential factors for selecting TPO-RAs, particularly in fasting. The administration of ROM is deemed valuable for individuals with ITP due to its ease of administration as a weekly subcutaneous injection without the need for any specific dietary or time restrictions. In Kuwait, medication is under a home administration program; therefore, adherence to the medication administration schedule is expected. However, some patients may prefer the oral route over injections; thus, AVA is considered a convenient option for patients, as it has no drug-food interaction and does not require dietary restrictions (35). A previous study showed that approximately 51% of patients switched from ELT to AVA due to its convenient administration without nutritional limits (36).

In alignment with the literature, our research demonstrated that the absorption of ELT is significantly influenced by food intake. The mean platelet count dropped significantly during Ramadan (*p* = 0.016). Consequently, it is essential to adhere to the administration’s instructions. Specifically, administering ELT with high-calcium food or an antacid containing aluminum and magnesium has resulted in significantly diminished systemic exposure (33).

In a recent study by Yassin et al., the authors proposed that alternating between TPO-RAs could be a beneficial strategy for fasting to circumvent obstacles in the administration route and dietary restrictions (37). ELT is not typically recommended due to potential drug-food interactions, and the limitations of nutritional restrictions and administration time during fasting pose additional challenges. Therefore, based on these findings, the use of AVA and ROM over ELT can be considered for ITP patients who wish to adopt an IF regimen.

The current study had several limitations that should be recognized. The major drawback is the inability to monitor the dietary intake of individuals, which is highlighted as a significant confounding factor in treatment with ELT. In addition, the small sample size and lack of controls are inevitable limitations of the current study, which may have affected the generalizability of the results. However, given that this was a multicenter study, it allowed for the enrollment of a diverse group of patients. Finally, although we attempted to verify compliance with treatment during Ramadan, we cannot rule out inadequate compliance.

## Conclusion

This study supports the feasibility of Ramadan fasting in ITP patients receiving TPO-RAs. The general tolerability and efficacy of TPO-RAs were reflected in the absence of major bleeding and the better efficacy of AVA and ROM, which were not affected by fasting. In concordance with the literature, our study showed that the absorption of ELT is highly affected by food consumption; thus, it is critical to adhere to the administration’s instructions. ROM showed better adherence to the administration schedule than ELT, contributing to its subcutaneous dosage form and the tailored home visit program provided weekly by trained nurses in Kuwait. These findings emphasize the need for healthcare providers to consider religious rituals and dietary habits when treating patients with ITP. Further studies with larger sample sizes are recommended to investigate the impact of other types of fasting on the efficacy and safety of TPO-RAs.

## Data Availability

The raw data supporting the conclusions of this article will be made available by the authors, without undue reservation.
